# Supplementary material for: L-DOPA modulates activity in the vmPFC, nucleus accumbens, and VTA during threat extinction learning in humans
Source: eLife. 2021 Sep 2;10:e65280. doi: 10.7554/eLife.65280 (PMC8443250; doi:10.7554/eLife.65280)
Supplement: Supplementary file 1. [file elife-65280-supp1.docx]

**Supplementary materials**

**Dopaminergic signals in the Nucleus Accumbens, VTA and vmPFC underpin extinction learning from the omission of expected threats**

**Supplementary File 1a: Testing for differences in US intensity and US valence ratings, maximums of ratings for the CS+ or CS- (two-sided unpaired t-tests).**

| **US Adjustment and evaluation** | | | | | | | | |
| --- | --- | --- | --- | --- | --- | --- | --- | --- |
| **condition** | | **measure** | | | **N** | **T** | **df** | **P** |
| Calibration | | US strenght (micro Ampere) | | | P N=22,  LD N=24 | 1.409 | 38.737 | .167 |
|  | | Rated US valence | | | P N=21,  LD N=24 | -.843 | 42.999 | .404 |
| Conditioning | | Rated US intensity | | | P N=22,  LD N=24 | -.279 | 43.769 | .782 |
|  | | CSs rating max (d1) | | | P N=22,  LD N=24 | -1.280 | 41.957 | .208 |
| Extinction | | CSs rating max (d2) | | | P N=22,  LD N=21 | -.845 | 40.733 | .403 |
|  |  | |  |  |  |  |  |  |
| Return of fear | | Rated US intensity | | | P N=22,  LD N=21 | 1.162 | 40.999 | .252 |
|  | | CSs rating max (d3) | | | P N=21,  LD N=24 | .525 | 40.852 | .603 |

Please note that US intensity was only calibrated on day 1. CS=conditioned stimulus, US=unconditioned stimulus, P=Placebo group, LD=L-DOPA group, T=T-values, df=degrees of freedom, P=p-values

**Supplementary File 1b: Results of the outcome measurements during acquisition training (repeated measures ANOVA)**

| **Fear acquisition training** | | | | | | | |
| --- | --- | --- | --- | --- | --- | --- | --- |
| **effect** | **measure** | **N** | **F** | **df**  **(GG)** | **P** | **Partial Eta^2^** | **Post-hoc (uncorr.)** |
| CS-type | Fear rating | P=22  LD=24 | 116.034 | 1,44 | < .001 | .725 | CS+ > CS-, < .001 |
|  |  |  |  |  |  |  |  |
|  | US-expectancy | P =22  LD =24 | 203.904 | 1,44 | < .001 | .823 | CS+ > CS-, < .001 |
|  |  |  |  |  |  |  |  |
|  | SCR | P=21  LD =24 | 41.734 | 1,43 | < .001 | .493 | CS+ > CS-, < .001 |
| CS-type * group | Fear rating | P=22  LD=24 | .046 | 1,44 | .831 | .001 |  |
|  |  |  |  |  |  |  |  |
|  | US expectancy | P=22  LD=24 | 1.390 | 1,44 | .245 | .031 |  |
|  |  |  |  |  |  |  |  |
|  | SCR | P=21  LD=24 | .179 | 1,43 | .674 | .004 |  |
| Time * group | Fear rating | P=22  LD=24 | 2.775 | 1,44 | .103 | .059 |  |
|  | US expectancy | P=22  LD=24 | .180 | 1.9,  84.6 | .827 | .004 |  |
|  | SCR | P=21  LD=24 | .331 | 1,43 | .709 | .008 |  |
| CS-type * time * group | Fear rating | P=22  LD=24 | .013 | 1,44 | .911 | < .001 |  |
|  | US expectancy | P=22  LD=24 | 3.307 | 1.9,  83.1 | .044 | .070 | **All CS by Block by group comparisons p>0.1, see Table S3** |
|  | SCR | P=21  LD=24 | .161 | 1,43 | .850 | .004 |  |
| group | Fear rating | P=22  LD=24 | .497 | 1,44 | .484 | .011 |  |
|  | US expectancy | P=22  LD=24 | .023 | 1,44 | .879 | .001 |  |
|  | SCR | P=21  LD=24 | .159 | 1,43 | .692 | .004 |  |

CS=conditioned stimulus, US=unconditioned stimulus, P=Placebo group, LD=L-DOPA group, T=T-values, df=degrees of freedom, P=p-values, unocrr.=uncorrected

**Supplementary File 1c: Post-hoc comparisons of CS-type * Time * group in US expectancy ratings. (Two-sided Independent Samples T-Test, since there is was hypothesis for day 1)**

|  | | | |  |
| --- | --- | --- | --- | --- |
|  | **t** | **df** | **p (uncorrected)** | **P(Holm- Bonferroni)** |
| Difference in US expectancy mean Block 1 | -0.060 | 44 | 0.953 | 0.953 |
| Difference in US expectancy mean Block 2 | 1.596 | 44 | 0.118 | 0.236 |
| Difference in US expectancy mean Block 3 | 1.761 | 44 | 0.085 | 0.255 |

| **Exploratory comparisons of CSs between groups** | | | |  |
| --- | --- | --- | --- | --- |
|  | **t** | **df** | **p (uncorrected)** | **P(Holm- Bonferroni)** |
| MEAN CS+ Block 1 | -0.099 | 44 | 0.922 | >0.99 |
| MEAN CS+ Block 2 | 1.204 | 44 | 0.235 | 0.705 |
| MEAN CS+ Block 3 | 1.634 | 44 | 0.109 | 0.654 |
| MEAN CS- Block 1 | -0.014 | 44 | 0.989 | 0.989 |
| MEAN CS- Block 2 | -1.310 | 44 | 0.197 | 0.788 |
| MEAN CS- Block 3 | -1.362 | 44 | 0.180 | 0.900 |

**Supplementary File 1d: Results of the outcome measurements during extinction training (repeated measures ANOVA)**

| **effect** | **measure** | **N** | **F** | **df (GG)** | **P** | **Eta^2^** | **Post-hoc (Holm-bonf. corrected)** |
| --- | --- | --- | --- | --- | --- | --- | --- |
| CS-type | Fear rating | P=22,  LD=21 | 61.830 | 1,41 | < .001 | .601 | CS+ > CS-, < .001 |
|  | US expectancy | P=22,  LD=21 | 22.327 | 1,41 | < .001 | .353 | CS+ > CS-, < .001 |
|  | SCR | P =20,  LD=20 | 23.861 | 1,38 | < .001 | .386 | CS+ > CS-, < .001 |
| Time | Fear rating | P =22,  LD=21 | 91.629 | 1, 41 | <0.001 | 0.691 | Block 1>  Block 2, p<0.001 |
|  | US expectancy | P =22,  LD=21 | 54.929 | 1.6, 61.5 | <.001 | .156 | Block 1> Block 2 >  Block 3, ps<0.001 |
|  | SCR | P =20,  LD=20 | 66.633 | 1.2, 47.3 | <.001 | .637 | Block 1> Block 2 >  Block 3, ps<0.001 |
| CS-type * time | Fear rating | P =22,  LD=21 | 50.081 | 1, 41 | <.001 | 0.550 | CS+ > CS- Block 1  p< .001,  CS+ > CS-Block 2, p= .103 |
|  | US expectancy | P =22,  LD=21 | 25.804 | 1.9, 72.2 | <.001 | .043 | CS+ > CS- Block 1  p< .001,  CS+ > CS-Block 2  p= .048,  CS+ > CS-Block 3  p=.57 |
|  | SCR | P =20,  LD=20 | 4.202 | 1.8, 66.6 | 0.023 | .100 | CS+ > CS- Block 1 p< .001,  CS+ > CS- Block 2 p= .026,  CS+ > CS- Block 3 p=.052 |
| CS-type * group | Fear rating | P =22,  LD=21 | 2.341 | 1,41 | .134 | .054 |  |
|  | US expectancy | P =22,  LD=21 | .001 | 1,41 | .977 | < .001 |  |
|  | SCR | P =20,  LD=20 | .003 | 1,38 | .955 | <.001 |  |
| Time * group | Fear rating | P =22,  LD=21 | .169 | 1,41 | .683 | 0.004 |  |
|  | US expectancy | P =22,  LD=21 | 1.392 | 1,41 | .253 | .033 |  |
|  | SCR | P =20,  LD=20 | 1.435 | 1,38 | .243 | .036 |  |
| CS-type * time * group | Fear rating | P =22,  LD=21 | 3.784 | 1,41 | .059 | .084 |  |
|  | US expectancy | P =22,  LD=21 | .367 | 1,41 | .667 | .009 |  |
|  | SCR | P =20,  LD=20 | .178 | 1,38 | .809 | .005 |  |
| group | Fear rating | P =22,  LD=21 | .0336 | 1,41 | .565 | .008 |  |
|  | US expectancy | P =22,  LD=21 | .001 | 1,41 | .972 | < .001 |  |
|  | SCR | P =20,  LD N=20 | 1.038 | 1,38 | .315 | .027 |  |

CS=conditioned stimulus, US=unconditioned stimulus, P=Placebo group, LD=L-DOPA group, T=T-values, df=degrees of freedom, P=p-values, GG=Greenhouse-Geisser

|  | **Supplementary File 1e: Group comparisons of differential (CS+ > CS-) fear ratings on day 2 between L-DOPA and Placebo (one-sided Independent Samples T-Test, L-DOPA > Placebo)** | | | | | | | | | | | | | |
| --- | --- | --- | --- | --- | --- | --- | --- | --- | --- | --- | --- | --- | --- | --- |
|  | | | **t** | | | **df** | |  | **P (uncorr)** | | **P(Holm- Bonferroni)** | | **Cohen's d** | |
| Differential rating CS+ > CS- pre | | | -1.911 |  | 41.000 | | 0.032 | | | 0.064 | | -0.583 | |  |
| Differential rating CS+ > CS- post | | | -0.246 |  | 41.000 | | 0.403 | | | 0.403 | | -0.075 | |  |
|  | | |  |  |  | |  | | |  | |  | |  |
| **Exploratory comparisons of CS and ITI responses between groups** | | | | | | | | | | | |  | |  |
| CS+ rating pre | | | -1.769 |  | 41.000 | | 0.042 | | | 0.256 | | -0.540 | |  |
| CS+ rating post | | | -0.659 |  | 41.000 | | 0.257 | | | >0.99 | | -0.201 | |  |
| CS- rating pre | | | 1.231 |  | 41.000 | | 0.887 | | | 0.887 | | 0.375 | |  |
| CS- rating post | | | -0.464 |  | 41.000 | | 0.322 | | | 0.966 | | -0.142 | |  |
| ITI (context) rating pre | | | -1.635 |  | 41.000 | | 0.055 | | | 0.257 | | -0.499 | |  |
| ITI (context) rating post | | | 0.611 |  | 41.000 | | 0.728 | | | >0.99 | | 0.186 | |  |
|  | | |  | | | | | |  | | | | | |
|  | | Note.For all tests, the alternative hypothesis specifies that L-DOPA group is less than Placebo | | | | | | | | | | | | |
|  | | |  | | | | | |  | | | | | |

We want to highlight that the range-correction of fear ratings (with the individual day-wise maximum) improved this difference between groups. As such, we found lower differential (non range-corrected) fear ratings during retrieval on day 2 in the L-DOPA group albeit lower support for statistical difference (one-sided, post-hoc independent t-test: L-DOPA<Placebo, t(41)=1.463, p(uncorr)=0.076, L-DOPA mean: 25.048 +/- 22.723 [SD], Placebo mean: 36.636 +/- 28.681 [SD]).

| **Supplementary File 1f Group comparisons between L-DOPA and Placebo for Pearce-Hall mean model estimates (Independent Samples T-Test)** | | | | | | | |
| --- | --- | --- | --- | --- | --- | --- | --- |
|  | | **t** | | **df** | | **P(uncorr)** | **P(Holm- Bonferroni)** |
| Prediction error |  | 0.097 |  | 39.993 |  | 0.923 | >0.99 |
| Associability |  | 0.015 |  | 39.998 |  | 0.988 | 0.988 |
| Learning rate |  | 0.179 |  | 39.383 |  | 0.859 | >0.99 |
| BIC |  | -0.411 |  | 34.299 |  | 0.684 | >0.99 |
| LLE |  | -0.411 |  | 34.299 |  | 0.684 | >0.99 |
| Value |  | -0.097 |  | 39.988 |  | 0.924 | >0.99 |
|  | | | | | | | |
|  | | | | | | | |
| Abbreviations:BIC= Bayesian information criterion, LLE =  Note.For all tests, the alternative hypothesis specifies that L-DOPA group is less than Placebo | | | | | | | |
|  | | | | | | | |

**Supplementary File 1g: Retrieval test**

| **Retrieval test** | | | | | | | |
| --- | --- | --- | --- | --- | --- | --- | --- |
| **effect** | **measure** | **N** | **F** | **df (GG)** | **P** | **Eta^2^** | **Post-hoc (Holm-bonf. corrected)** |
| CS-type | Fear rating | P N=21,  LD N=21 | 54.79 | 1,40 | < .001 | .578 | CS+ > CS-, < .001 |
|  |  |  |  |  |  |  |  |
|  | US expectancy | P N=22,  LD N=21 | 15.172 | 1,41 | < .001 | .270 | CS+ > CS-, < .001 |
|  |  |  |  |  |  |  |  |
|  | SCR | P N=21,  LD N=21 | 24.071 | 1,40 | < .001 | .122 | CS+ > CS-, < .001 |
| CS-type * group | Fear rating | P N=21,  LD N=21 | 1.229 | 1,40 | .274 | .030 |  |
|  |  |  |  |  |  |  |  |
|  | US expectancy | P N=22,  LD N=21 | .010 | 1,41 | .921 | .001 |  |
|  |  |  |  |  |  |  |  |
|  | SCR | P N=21,  LD N=21 | .005 | 1,40 | .496 | .002 |  |
| group | Fear rating | P N=21,  LD N=21 | .233 | 1,41 | .632 | .006 |  |
|  | US expectancy | P N=22,  LD N=21 | .892 | 1,41 | .351 | .021 |  |
|  | SCR | P N=20,  LD N=20 | 0.027 | 1,40 | .870 | .001 |  |

CS=conditioned stimulus, US=unconditioned stimulus, P=Placebo group, LD=L-DOPA group, T=T-values, df=degrees of freedom, P=p-values, GG=Greenhouse-Geisser

**Reinstatement analyses**

**Supplementary File 1h: Reinstatement test (block 1 vs block 2)**

| **Reinstatement (blockwise comparisions)** | | | | | | | |
| --- | --- | --- | --- | --- | --- | --- | --- |
| **effect** | **measure** | **N** | **F** | **df (GG)** | **P** | **Eta^2^** | **Post-hoc (Holm-bonf. corrected)** |
| CS-type | US expectancy | P N=22,  LD N=21 | 20.674 | 1,41 | < .001 | .148 | CS+ > CS-, < .001 |
|  |  |  |  |  |  |  |  |
|  | SCR | P N=21,  LD N=21 | 31.367 | 1,40 | < .001 | .440 | CS+ > CS-, < .001 |
| Reinstatement |  |  |  |  |  |  |  |
|  | US expectancy | P N=22,  LD N=21 | 19.151 | 1,41 | <.001 | .027 | Block 1>  Block 2  ps<0.001 |
|  | SCR | P N=21,  LD N=21 | 19.786 | 1, 40 | <.001 | .331 | Block 1>  Block 2, <0.001 |
| CS-type * reinstatement |  |  |  |  |  |  |  |
|  | US expectancy | P N=22,  LD N=21 | 8.222 | 1,41 | <.001 | .011 | CS+ Block 1>  CS+ Block 2 p< .001 |
|  | SCR | P N=21,  LD N=21 | 3.989 | 1,40 | 0.053 | .091 | CS+ Block 1>  CS+ Block 2, p< .001 |
| CS-type * group |  |  |  |  |  |  |  |
|  | US expectancy | P N=22,  LD N=21 | .042 | 1,41 | .838 | < .001 |  |
|  |  |  |  |  |  |  |  |
|  | SCR | P N=21,  LD N=21 | 1.073 | 1,40 | .306 | .026 |  |
| Reinstatement * group |  |  |  |  |  |  |  |
|  | US expectancy | P N=22,  LD N=21 | 0.174 | 1,41 | .678 | <.001 |  |
|  | SCR | P N=21,  LD N=21 | 0.002 | 1,40 | .965 | <.001 |  |
| CS-type * reinstatement * group |  |  |  |  |  |  |  |
|  | US expectancy | P N=22,  LD N=21 | .103 | 1,41 | .750 | <.001 |  |
|  | SCR | P N=21,  LD N=21 | .004 | 1,40 | .951 | <.001 |  |
| group |  |  |  |  |  |  |  |
|  | US expectancy | P N=22,  LD N=21 | .506 | 1,41 | .481 | .012 |  |
|  | SCR | P N=21,  LD N=21 | 1.038 | 1,40 | .867 | <.001 |  |

CS=conditioned stimulus, US=unconditioned stimulus, P=Placebo group, LD=L-DOPA group, T=T-values, df=degrees of freedom, P=p-values, GG=Greenhouse-Geisser

**Supplementary File 1i: Reinstatement test (average across 3 trials before vs. after the reinstatement procedure)**

| **Reinstatement (3 trials)** | | | | | | | |
| --- | --- | --- | --- | --- | --- | --- | --- |
| **effect** | **measure** | **N** | **F** | **df (GG)** | **P** | **Eta^2^** | **Post-hoc (Holm-bonf. corrected)** |
| CS-type | US expectancy | P N=22,  LD N=21 | 13.541 | 1,41 | < .001 | .248 | CS+ > CS-, < .001 |
|  |  |  |  |  |  |  |  |
|  | SCR | P N=21,  LD N=21 | 14.486 | 1,40 | < .001 | .226 | CS+ > CS-, < .001 |
| Reinstatement |  |  |  |  |  |  |  |
|  | US expectancy | P N=22,  LD N=21 | 5.360 | 1,41 | .026 | .116 | Block 1>  Block 2, p<0.001 |
|  | SCR | P N=21,  LD N=21 | 4.136 | 1,40 | .004 | .094 | Block 1>  Block 2, <0.001 |
| CS-type * reinstatement |  |  |  |  |  |  |  |
|  | US expectancy | P N=22,  LD N=21 | 1.641 | 1,41 | .207 | .038 |  |
|  | SCR | P N=21,  LD N=21 | 0.420 | 1,40 | .521 | .010 |  |
| CS-type * group |  |  |  |  |  |  |  |
|  | US expectancy | P N=22,  LD N=21 | .047 | 1,41 | .829 | <.001 |  |
|  |  |  |  |  |  |  |  |
|  | SCR | P N=21,  LD N=21 | 5.443 | 1,40 | .025 | .120 | See Table S8 for group comparisons |
| Reinstatement * group |  |  |  |  |  |  |  |
|  | US expectancy | P N=22,  LD N=21 | 2.254 | 1,41 | .141 | .052 |  |
|  | SCR | P N=21,  LD N=21 | .020 | 1,40 | .880 | .001 |  |
| CS-type * reinstatement * group |  |  |  |  |  |  |  |
|  | US expectancy | P N=22,  LD N=21 | 1.113 | 1,41 | .207 | .038 |  |
|  | SCR | P N=21,  LD N=21 | 1.192 | 1,40 | .282 | .029 |  |
| group |  |  |  |  |  |  |  |
|  | US expectancy | P N=22,  LD N=21 | .159 | 1,41 | .692 | .004 |  |
|  | SCR | P N=21,  LD N=21 | <.001 | 1,40 | .978 | <.001 |  |

CS=conditioned stimulus, P=Placebo group, LD=L-DOPA group, T=T-values, df=degrees of freedom, P=p-values, GG=Greenhouse-Geisser

| **Supplementary File 1j: CS-type by group comparisons (one-sided Independent Samples T-Test, L-DOPA<Placebo)** | | | | | | | |
| --- | --- | --- | --- | --- | --- | --- | --- |
| Differential response (CS+>CS-) | | **t** | | **df** | | **p (holm-**  **bonf. corrected)** | |
| Mean 3 trials before Reinstatement |  | -0.735 |  | 40.000 |  | 0.233 |  |
| Mean 3 trials after Reinstatement |  | -2.405 |  | 40.000 |  | 0.020 |  |
|  | | | | | | | |
|  | | | | | | | |
| Note.For all tests, the alternative hypothesis specifies that L-DOPA group is less than Placebo | | | | | | | |
|  | | | | | | | |

**Control analyses for the reinstatement test (average across 3 trials before vs. after the reinstatement procedure)**

During the review process, it was suggested to test if CS+ or CS- responses (average across 3 trials) after reinstatement were different between groups. We found no support for a difference between groups when testing two-tailed (in order to test for differences in general between groups).

| **Supplementary File 1k: CS specific group comparisons after reinstatement**  **(two-sided Independent Samples T-Test)** | | | | |  |  |  |
| --- | --- | --- | --- | --- | --- | --- | --- |
| Response | | **t** | | **df** | | **p (uncorrected)** | |
| CS+ after Reinstatement |  | -1.348 |  | 40 |  | 0.185 |  |
| CS- after Reinstatement |  | 1.288 |  | 40 |  | 0.205 |  |
|  | | | | | | | |
|  | | | | | | | |
|  | | | | | | | |

Additionally, we tested how the analyses of group differences in reinstatement change as a function of trials that are included in these analyses. In general, our analyses show that we found an interaction with the factor group for all analyses (see **Supplementary File 1i**). Furthermore, we found support for lower differential responses in the L-DOPA group, as compared to Placebo after reinstatement (see **Supplementary File 1j**). Additionally, we illustrated the effect size of comparisons of differential CS responses between groups (one-sided unpaired t- before and after reinstatement) as a function of trials, which indicates a medium effect size for comparisons after reinstatement when averaging across 2 to 4 trials.

**Supplementary File 1l: Reinstatement analyses (rmANOVA) as a function of trials before and after reinstatement**

|  | **CS-type** | | | **Reinstatement** | | | **CS*Group** | | | **CS*Group*Reinst** | | |
| --- | --- | --- | --- | --- | --- | --- | --- | --- | --- | --- | --- | --- |
|  | **F** | **p** | **η² _p_** | **F** | **p** | **η² _p_** | **F** | **p** | **η² _p_** | **F** | **p** | **η² _p_** |
| **2** | 5.428 | **0.025** | 0.119 | 8.808 | **0.005** | 0.180 | 2.045 | 0.160 | 0.049 | 4.360 | **0.043** | 0.098 |
| **3** | 14.486 | **<0.001** | 0.266 | 1.668 | 0.204 | 0.040 | 5.443 | **0.025** | 0.120 | 1.192 | 0.282 | 0.029 |
| **4** | 20.312 | **<0.001** | 0.337 | 2.026 | 0.162 | 0.048 | 5.767 | **0.021** | 0.126 | 0.387 | 0.537 | 0.010 |
| **5** | 27.683 | **<0.001** | 0.409 | 0.275 | 0.603 | 0.007 | 3.370 | **0.074** | 0.409 | 0.017 | 0.896 | <0.001 |

**Supplementary File 1m: Comparisons of differential CS responses between groups (one-sided unpaired t- before and after reinstatement**

| **p-value** | **bf** |  |  | **after** |  |  |
| --- | --- | --- | --- | --- | --- | --- |
|  | **Diff CS** | **CS+** | **CS-** | **Diff CS** | **CS+** | **CS-** |
| **2** | 0.718 | 0.514 | 0.291 | **0.017** | 0.133 | 0.942 |
| **3** | 0.233 | 0.390 | 0.686 | **0.010** | **0.093** | 0.897 |
| **4** | 0.126 | 0.417 | 0.838 | **0.023** | 0.166 | 0.877 |
| **5** | 0.114 | 0.444 | 0.846 | **0.069** | 0.294 | 0.795 |

For all tests, the alternative hypothesis specifies that group *1(L-DOPA)* is less than group *2 (Placebo).* Please note that p-values in this table have not been corrected for multiple comparisons, but are corrected for the main analyses (3 trials) in the manuscript. Diff CS= CS+ - CS-

**Supplementary File 1n: Additional analyses of nucleus accumbens activity during acquisition and extinction training (one-sided full factorial models of parameter estimates)**

|  | **CS+ Acq** | **CS+ >CS- Acq** | **US Acq** | **CS+ Ext** |
| --- | --- | --- | --- | --- |
| **Whole sample** | Left  XYZ [MNI]= -12 15 -4  P(FWE)= 0.086  T= 2.75  P(uncorr)= 0.005  Right  XYZ [MNI]= 12 10 -9  P(FWE)= 0.005  T= 3.93  P(uncorr)< 0.001 | Left  XYZ [MNI]= -10 12 -8  P(FWE)= 0.061  T=2.90  P(uncorr)= 0.003  Right  XYZ [MNI]= 12 10 -10  P(FWE)= 0.004  T=3.98  P(uncorr) < 0.001 | Left  NO SUPRA- THRESHOLD CLUSTERS  Right  XYZ [MNI]= 9 6 -8  P(FWE)= 0.018  T=3.39  P(uncorr)= 0.001  (only 2 voxels) | Left & Right  NO SUPRA- THRESHOLD CLUSTERS |
| **L-DOPA> Placebo** | Left  NO SUPRA-THRESHOLD CLUSTERS  Right  XYZ [MNI]= 10 11 -7  P(FWE)= 0.086  T=2.81  P(uncorr)= 0.004 | Left & Right  NO SUPRA-THRESHOLD CLUSTERS | Left & Right  NO SUPRA-THRESHOLD CLUSTERS | Left & Right  NO SUPRA-THRESHOLD CLUSTERS |

Acq =Acquisition training, Ext=Extinction training, MNI=Montreal Neurological Institute CS=conditioned stimulus, US=unconditioned stimulus Placebo=Placebo group, L-DOPA=L-DOPA group, T=T-values, P=p-values, FWE=family-wise error correction for independent voxels within the region of interest.

**Supplementary File 1o: Additional psychophysiological interaction analysis during acquisition and extinction training (one-sided independent t-test models on connectivity estimates)**

|  | **CS+ Acq** | **US Acq** | **CS+ Ext** |
| --- | --- | --- | --- |
| **Connectivity**  **Nucleus Accumbens –**  **VTA**  **(whole sample)** | XYZ [MNI]= -3 -18 -16  P(FWE)= 0.064  T=3.53  P(uncorr)=0.001 | P(FWE)=0.279 | **---** |
| **Connectivity**  **Nucleus Accumbens –Amygdala**  **(whole sample)** | Left  NO SUPRATHRESHOLD CLUSTERS  Right  P(FWE)=0.304 | Left  NO SUPRATHRESHOLD CLUSTERS  Right  P(FWE)=0.279 |  |
| **Connectivity**  **Nucleus Accumbens**  **(L-DOPA> Placebo)** | VTA  P(FWE)> 0.264  Amygdala (L&R)  P(FWE)>0.122 | VTA  P(FWE)>0.210  Amygdala (L&R)  P(FWE)>0.111 | NO SUPRATHRESHOLD CLUSTERS |

Acq =Acquisition training, Ext=Extinction training, MNI=Montreal Neurological Institute CS=conditioned stimulus, US=unconditioned stimulus Placebo=Placebo group, L-DOPA=L-DOPA group, T=T-values, P=p-values, FWE=family-wise error correction for independent voxels within the region of interest.
